# Involvement of Type IV Pili in Pathogenicity of Plant Pathogenic Bacteria

**DOI:** 10.3390/genes2040706

**Published:** 2011-10-18

**Authors:** Saul Burdman, Ofir Bahar, Jennifer K. Parker, Leonardo De La Fuente

**Affiliations:** 1 Department of Plant Pathology and Microbiology and the Otto Warburg Center for Agricultural Biotechnology, The Robert H. Smith Faculty of Agriculture, Food and Environment, The Hebrew University of Jerusalem, Rehovot 76100, Israel; 2 Department of Entomology and Plant Pathology, Auburn University, Auburn, AL 36849, USA; E-Mails: jkp0006@auburn.edu (J.K.P.); lzd0005@auburn.edu (L.D.L.F.)

**Keywords:** type IV pili, twitching, virulence, biofilm

## Abstract

Type IV pili (T4P) are hair-like appendages found on the surface of a wide range of bacteria belonging to the β-, γ-, and δ-Proteobacteria, Cyanobacteria and Firmicutes. They constitute an efficient device for a particular type of bacterial surface motility, named twitching, and are involved in several other bacterial activities and functions, including surface adherence, colonization, biofilm formation, genetic material uptake and virulence. Tens of genes are involved in T4P synthesis and regulation, with the majority of them being generally named *pil/fim* genes. Despite the multiple functionality of T4P and their well-established role in pathogenicity of animal pathogenic bacteria, relatively little attention has been given to the role of T4P in plant pathogenic bacteria. Only in recent years studies have begun to examine with more attention the relevance of these surface appendages for virulence of plant bacterial pathogens. The aim of this review is to summarize the current knowledge about T4P genetic machinery and its role in the interactions between phytopathogenic bacteria and their plant hosts.

## 1. Introduction

Pili or fimbriae are hair-like appendages found on the surface of many bacteria. There are several types of pili, which differ in their mechanisms of assembly, structure and function. Among them, type IV pili (T4P) are widespread among diverse members of the β-, γ-, and δ-Proteobacteria, Cyanobacteria and Firmicutes. T4P are therefore found in both Gram-negative and Gram-positive bacteria, supporting an ancient origin for this kind of pili [1,2]. T4P are proteinaceous, flexible filaments with a diameter of 5–8 nm, which extend up to several micrometers in length. They are generally located at one or both poles of a cell, where they mediate twitching motility, an efficient and versatile flagellar-independent form of bacterial surface motility [3,4] (supplementary movie 1). T4P have also been shown to be involved in a variety of other bacterial activities and features, including surface adhesion and colonization, biofilm formation, genetic material uptake and virulence [1,5].

*Pseudomonas aeruginosa* has been the principal model for investigation of genetic and functional aspects of T4P. *P. aeruginosa* can be easily cultured and molecularly manipulated, and there is a strong basis of genetic and genomic approaches available for this bacterium. In addition, twitching motility can be easily scored in this bacterium by the naked eye. Importantly, *P. aeruginosa* is an opportunistic pathogen, being the main causal agent of lung damage in cystic fibrosis patients and responsible for severe infections in immunocompromised individuals. It is also able to cause disease in a wide range of animals and plants [4,6,7]. Other well-studied bacterial species in which T4P have been thoroughly investigated are *Myxococcus xanthus, Neisseria gonorrhoeae* and *Neisseria meningitidis. M. xanthus* is a model organism for studying bacterial social behavior and development (specifically fruiting body formation), features that are strongly associated with T4P [4,8-10]. The above *Neisseria* species are important human pathogens and possess a high natural competence for transformation [4,11].

**Figure 1:**
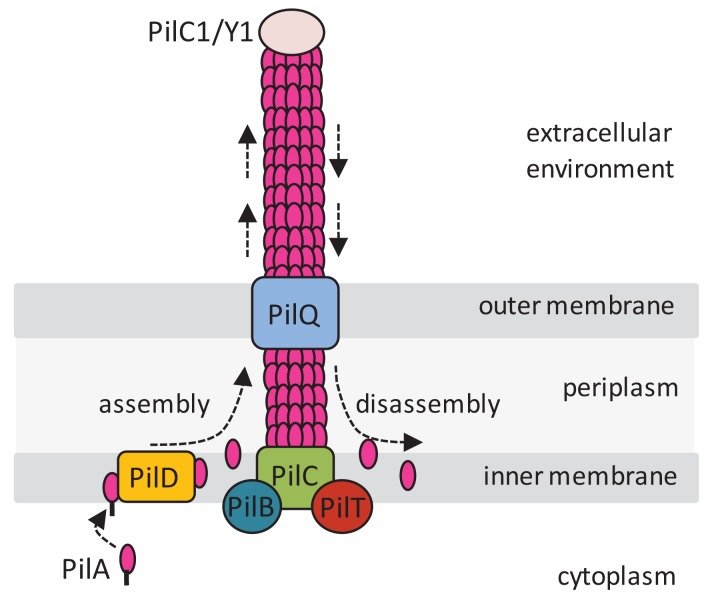
Type IV pili (T4P) structure and function. The T4P filament is mainly composed of pilin (PilA) subunits that are synthesized as prepilin and cleaved by the action of PilD, which also methylates *N*-terminal phenylalanine of the mature pilin. PilA units are assembled into the pilus by the cytoplasmic membrane protein PilC, with the filament emerging out via the outer membrane secretin PilQ. The ATPase proteins PilB and PilT mediate pilus assembly (extension) and disassembly (retraction), respectively. Proteins are named according to the *Pseudomonas aeruginosa* nomenclature, which is the one generally used for plant pathogenic bacteria (see text and Table 1 for functions of other proteins and alternative nomenclatures). This figure was prepared based on a figure from Chen and Dubnau [13].

T4P are mainly composed of thousands of copies of a small (13–23 kDa) subunit named pilin (in most cases termed PilA). All type IV pilins are synthesized as prepilins, which are processed by a peptidase that removes their *N*-terminal leader peptide (Figure 1). Based on differences in the length of their leader peptides and mature sequences, type IV pilins are divided into two subgroups, type IVa and type IVb. Type IVa pilins possess shorter leader peptides (less than 10 aa) and a typical length of about 150-160 aa. In contrast, type IVb pilins exhibit longer leader peptides (about 15–30 aa), and are either longer (180-200 aa) or shorter (about 40–50 aa) than type IVa pilins [5]. Interestingly, while type IVa pilins are present in a broad range of bacteria, type IVb pilins are found almost exclusively in enteric pathogens like *Vibrio cholerae*, enteropathogenic and enterotoxigenic *Escherichia coli* and *Salmonella enterica* [12].

The secondary structure of pilin, as well as its structure in the pilus filament, was first reported in 1995 for *N. gonorrhoeae* by Parge and colleagues [14]. Despite a limited sequence similarity beyond the first 25 aa residues, type IV pilins share a common architecture, being mainly characterized by a highly hydrophobic *N*-terminal region of ∼60 residues arranged in α-helices and a more variable and hydrophilic C-terminal region, mainly organized as 4 or 5 anti-parallel stranded-β-sheets. The *N*-terminal regions of adjacent monomers form an α-helical coiled-coil structure that is tucked inside the pilus fiber and covered by the *C*-terminal regions of adjacent monomers, which form a scaffold of β-strands. Although type IVa and type IVb pilins share a similar architecture, they differ in their β-sheet topology [2]. While most genes involved in biogenesis and regulation of type IVa pili are named *pil* genes, the gene nomenclature for type IVb pili is not conserved among different bacteria (Table 1).

**Table 1:** Core components of T4P structure and function according to the *Pseudomonas aeruginosa* nomenclature. Some of the alternative names of homologous proteins in other bacteria are also indicated.

**Protein**	**Function**	**Alternative names a**
**PilA**	Pilin, main component of the T4P filament	PilE b, FimA c
**PilB**	ATPase that mediates pilus assembly and extension	PilF b, FimN c
**PilC**	Inner membrane protein	PilG b, FimO c
**PilD**	Prepilin leader peptidase/pilin *N*-terminus methylase	FimP c
**PilQ**	Outer membrane secretin	ComE d
**PilT**	ATPase that mediates pilus retraction and disassembly	PilU e

a Several alternative names are shown for components of type IVa pili. The nomenclature of type IVb proteins is not conserved and is not indicated here. For such information, see the review of Craig and Li [12];

b *Neisseria* spp.;

c Dichelobacter nodosus;

d *Haemophilus influenzae*;

e Pseudomonas aeruginosa.

In the following sections, we briefly describe the bacterial functions and activities that have been associated with T4P and the genetic machinery involved in its biogenesis and regulation. Next, we summarize the current knowledge of the role played by T4P in virulence and fitness of plant pathogenic bacteria. The structure of the T4P apparatus, as well as the complex mechanisms by which T4P assemble and disassemble, were reviewed recently [1,2,12,15] and will therefore not be addressed in detail in this review.

## 2. Features Associated with T4P

### 2.1. Twitching Motility

The term “twitching motility” was first introduced by Lautrop in 1961 to describe a type of flagellar-independent bacterial surface motility [16]. However, it was in the early 1970s that the association between twitching motility and T4P started to be elucidated by Henrichsen and colleagues. They showed that in *Moraxella* spp., *Acinetobacter* spp. and other Gram-negative strains, twitching motility strongly correlates with the formation of polar fimbriae, which later were termed type IV pili/fimbriae [17-19]. Henrichsen studied twitching motility on moist solid media and defined twitching motility as a type of surface translocation that produces spreading zones at the colony edges [20]. These twitching zones can be formed by one or several layers of cells, depending on the bacterial strain and growth conditions. Similar findings on the association between surface motility and pili were reported in 1976 by MacRae and McCurdy for *M. xanthus* [21], in which this type of motility is referred to as “gliding”.

Microscopic studies with non-piliated and hyperpiliated mutants of *P. aeruginosa* by Bradley in 1980 confirmed that T4P are required for twitching motility and evidenced that fimbrial extension and retraction is the mechanical basis of this movement [22]. Essentially, the distal end of T4P adheres to the surface and then retraction from the base of the pilus provides the force that pulls the cell forward. Eight years earlier, Bradley provided the first evidence of the role of T4P retraction in phage invasion. He showed that bacteriophage PP7 attaches to the tip of the *P. aeruginosa* pilus, and the average length of the pilus is significantly reduced upon incubation of the bacterium with the phage, bringing the phage into contact with the bacterial cell surface [23,24].

Strong evidence for pilus retraction was reported in 2000 for *N. gonorrhoeae* T4P by Merz and colleagues, who measured the velocity, timing and force of pilus retraction [25]. The retraction velocity was about 1.2 μm s^−1^, a value that correlated with the observed cell motility rates (about 1 μm s^−1^), supporting the idea that pilus retraction is responsible for twitching motility in this bacterium. Observations consistent with the association between T4P retraction and cell movement were reported in the same year for *M. xanthus* in studies comparing wild type and T4P mutants of this bacterium [26]. These findings were further supported by studies with *P. aeruginosa* by Skerker and Berg, who used a fluorescent dye to label T4P, thus allowing microscopic visualization of pili in live bacteria [27]. This study revealed that, similar to what was inferred for *N. gonorrhoeae* and *M. xanthus*, T4P in *P. aeruginosa* retract, and pilus retraction is responsible for pulling the cell forward. Here too, the velocity of pilus retraction correlated with that of cell movement (∼0.5 and ∼0.3 μm s^−1^, respectively). In addition, it was shown that pilus extension is not associated with cell movement, indicating that T4P can pull but not push. The mechanism by which T4P generate twitching motility was reviewed recently by Burrows [15].

### 2.2. Adhesive Properties and Biofilm Formation

No matter the lifestyle of a given bacterium, the ability to adhere to surfaces and to other cells is a crucial property, as it allows bacteria to efficiently colonize different niches, often through formation of biofilms. Biofilms are the most common mode of bacterial growth in nature and can be defined as communities of microorganisms attached to a surface [28]. Substantial evidence has accumulated since the mid-1980s on the adhesive properties of T4P and their importance for biofilm formation in *P. aeruginosa* and other bacteria [28-34]. Indeed, O'Toole and Kolter [35] identified several *pil*^−^ mutants of *P. aeruginosa* in a screen for defective biofilm formation ability. The *pil*^−^ mutants were shown to poorly attach to a plastic surface relative to the wild type, and they were unable to move and form cell aggregates. In agreement with these results, Heydorn and colleagues showed that *P. aeruginosa pil*^−^ mutants were severely affected in biofilm formation in a flow chamber relative to wild-type cells [36].

T4P functionality, particularly pilus retraction, is generally important for the adhesive properties conferred by these filaments. T4P retraction is necessary, not only for twitching motility, but also for formation of microcolonies in *N. gonorrhoeae* [25]. T4P retraction requires a protein that possesses ATPase activity, generally named PilT. *pilT* mutants are able to produce T4P; however, these mutants are often hyperpiliated [33,37] and lack pilus retraction and twitching abilities. In *N. gonorrhoeae* and *M. xanthus, pilT* mutants are able to tether to inert surfaces and to other cells, but they are unable to form microcolonies or a wide raft of cells, respectively [25,38]. In *P. aeruginosa*, under static conditions, *pilT* mutants form a much denser biofilm than the wild type; however, under flow conditions, these mutants produce biofilm mushroom-like structures that are less dense than those produced by the wild type [33]. A *pilA* mutant of this bacterium, impaired in pilin synthesis, was severely affected in biofilm formation under static conditions and was not able to form biofilms at all under flow conditions [33]. In agreement with these results, Klausen and colleagues showed that, in *P. aeruginosa*, twitching motility is required for the development of biofilm mushroom-like caps [39]. Similarly, in *M. xanthus*, T4P-mediated motility was shown to be required for normal fruiting body formation [10]. Recently, Conrad and colleagues showed that in *P. aeruginosa*, T4P interact cooperatively with flagella to influence the biofilm morphology through changes in movement orientation [40].

### 2.3. Virulence

The ability to adhere to the host cell surface is an important requisite for pathogenicity, as this step is required to initiate an infection. Several studies with various animal pathogenic bacteria have shown that bacteria that have lost T4P possess reduced adhesion and host colonization abilities, which are often associated with reduced virulence or lack of pathogenicity depending on the system investigated [29-32,41-43]. In many Gram-negative pathogenic bacteria, type III secretion systems (T3SS) play an important role in virulence by injecting effector proteins into eukaryotic host cells where they promote disease [44,45]. In *P. aeruginosa*, expression of T3SS-related genes was shown to be induced by bacterial contact to host cells mediated by T4P [46]. Moreover, global regulatory systems are known to promote virulence in this bacterium through mediation of toxin production, biosynthesis of flagella and T4P, and type III secretion [47,48]. Recently it has been shown that common two-component regulatory systems regulate the expression of T4P and multidrug resistance-related genes, revealing an intricate regulatory network for pili formation and antibiotic resistance [49]. Importantly, T4P are able to elicit host immune responses, and vaccines produced with purified pili can provide protection against infection with serologically related strains [4,50,51].

For several pathogenic bacteria, the contribution of T4P to virulence depends not only on the presence of T4P but also on their retraction ability. *pilT* mutants of *P. aeruginosa* and *N. gonorrhoeae* as well as *bfpF* (*pilT* homolog) of enteropathogenic *E. coli* display a significant reduction (or even lack) of cytotoxicity [43,52-54]. As mentioned, *pilT* mutants generally produce T4P but lack pilus retraction and twitching ability. As detailed in section 4 of this review, functional T4P are also required for wild-type levels of virulence in several plant pathogenic bacteria.

### 2.4. DNA Uptake and Protein Secretion

Several genes required for T4P assembly are homologous to genes involved in type II protein secretion and DNA uptake systems, indicating that these systems share a common evolutionary origin and architecture [4,55]. Homologous genes in these systems encode several components including pilins and pseudopilins, prepilin-processing leader peptidases, ATPases, multi-spanning transmembrane proteins and outer-membrane secretins [7,55]. Interestingly, in *P. aeruginosa*, PilA is required for optimal protein secretion [56]. T4P are strongly associated with competence for DNA transformation in many bacteria, thus this type of pilus is thought to play an important role in horizontal gene transfer [4,55]. Due to their high natural competence for DNA transformation, *Neisseria* species have been the principal model organisms for investigation of the association between transformation competence and T4P [11,57].

The mechanism by which T4P mediate DNA transformation has not been elucidated. Nevertheless, experimental evidence from several studies indicates that some of the proteins involved in pilus assembly and disassembly systems, but not the pilus structures *per se*, are essential for DNA translocation [55]. In support of this notion, the space formed within the core of the T4P filament is not sufficiently wide to allow nucleic acid passage [4]. Moreover, several naturally transformable bacteria, such as *Bacillus subtilis*, *Streptococcus pneumoniae* and *Haemophilus influenzae* do not produce T4P, although their transformation machineries comprise components that are similar to pilins and are required for transformation competence [55].

## 3. Genes Involved in T4P Synthesis and Variability of Pilin

### 3.1. Core Components of the T4P Machinery

Several recent reviews have described the current knowledge about genes involved in T4P synthesis and regulation [3,4,12,15]; therefore, these aspects will not be addressed in detail in this review. In *P. aeruginosa*, over 40 genes have been shown to be involved in T4P assembly, function and regulation [4,7,58]. The biogenesis and function of T4P are controlled by multiple signal transduction systems, including two-component regulatory systems [59-61], global carbon metabolism regulators [62], chemosensory systems [26,63-67] and quorum sensing [68,69]. In many bacterial species, the core genes involved in T4P assembly are located in the same operon and encode key conserved components (Table 1) that have homologs in type II secretion and archaeal flagellar systems [5,70-72]. For consistency and clarity, here we use the terminology used in *P. aeruginosa*, which is the one adopted for most plant pathogenic bacteria.

Some of the key protein components necessary for T4P synthesis and function, known to be conserved among bacterial species, are shown in Figure 1. These include: (i) PilA, the pilin subunit that constitutes the main structural component of T4P; (ii) PilD, a peptidase/N-methylase situated in the inner membrane of the bacterial cell. PilD recognizes the conserved *N*-terminal region of PilA, cleaves the leader peptide of prepilin proteins in a step that is necessary for T4P assembly [73]. PilD also catalyzes *N*-methylation of the amino-terminal phenylalanine of mature pilin [74], and cleaves other prepilin-like leader sequences of proteins involved in secretion [73]; (iii) PilB and PilT proteins, which are necessary for filament extension/polymerization and retraction, respectively [1,75]. Both PilB and PilT are ATPases that function as the motor responsible for twitching motility and are located in the cell inner membrane, or periplasm (PilB) and cytoplasm (PilT) [1]. PilT mutants are generally hyperpiliated, non-motile [76], and resistant to pilus-specific bacteriophages [77]. Mutations in PilB abolish production of T4P [76]. PilB and PilT are very well conserved among bacteria [78]; (iv) PilQ, an outer membrane secretin that is involved in export of pilin subunits [79]. Mutagenesis analyses in *pilQ* of *N. gonorrhoeae* indicate that this protein may have multiple roles related to pilus extrusion and function and host adherence [79].

### 3.2. Variability in Pilin Sequence

Pilin (PilA) is a logical choice for studies of diversity, since it is the main structural subunit of T4P. As mentioned, besides the highly conserved first 25 aa residues of the *N*-terminus, PilA proteins show limited sequence similarity [12]. Nevertheless, PilA proteins share a conserved structure among bacteria. They are small proteins of 13–23 kDa, consisting of a hydrophobic *N*-terminal region (∼60 residues) arranged in α-helices and a hydrophilic *C*-terminal region, mainly organized as β-sheets [2,14,15]. At the *C*-terminus, PilA has two cysteine residues that create a conserved disulfide-bonded loop (DSL) of variable size between 12 and 31 amino acids [80]. The DSL is believed to be exposed at the pili surface where it interacts with eukaryotic glycolipid receptors [80] and is proposed to have a role in adherence. Amino acids inside the DSL have key roles in T4P assembly and twitching motility [81].

Pilin sequences in *Neisseria* species (termed PilE) undergo great antigenic variation, although they were recently shown to be conserved among specific clonal complexes of *N. meningitidis* [82]. Due to sequence variation, pilin has been discarded as an optional vaccine target for *Neisseria* spp. Studies on population diversity among isolates of *P. aeruginosa* [80], defined five phylogenetic groups based on T4P sequences, particularly focusing on the pilin gene. It was found that one group was more prevalent in cystic fibrosis patients and environmental samples, as opposed to other types of clinical samples. This work showed a strong relationship between a specific pilin allele in *P. aeruginosa* and cystic fibrosis patients, indicating a possible role of T4P in host specificity. Moreover, a specific *pilA* variant was associated with isolates causing keratitis [83], further supporting the role of this gene in disease development. In another study, *pilA* was used to divide the human pathogen *Moraxella catharralis* into two clades based mainly on DNA sequence divergence at the *C*-terminus [84]. Isolates from both clades did not differ in pathogenicity despite the diverse origins of isolation from different patients and geographic locations [84]. The authors concluded that, for this bacterium, this highly conserved gene is a good candidate as an antigen for vaccine development.

Few studies have dealt with variability of gene sequences and structures of *pil* genes other than the pilin-encoding *pilA.* Among the few exceptions are recent studies of *N. gonorrhoeae pilQ* divergence among clinical isolates. The authors found nine groups differing in amino acid sequence among the tested population, but could not find a correlation with differences in antibiotic resistance [85].

### 3.3. Pilin Sequence Variability among Plant Pathogenic Bacteria

Some studies on sequence variability of pilin genes have been performed with animal pathogens, and these are primarily restricted to studies of pathogens within the same genus or species. To our knowledge, there are no previous studies addressing the variability of pilin *(pilA*) genes among plant pathogenic bacteria or comparing pilin gene sequences from plant and animal pathogenic bacteria. For this review, we conducted phylogenetic analyses of 71 pilin gene sequences (usually annotated as *pilA)* from plant and mammalian bacterial pathogen sequences available in GenBank (Table S1, supplementary material). Gene sequences were acquired only from complete genome sequences of well-known Gram-negative bacterial pathogens with satisfactory annotation, and the pilin gene designation was confirmed by BLAST. For alleles present in multiple isolates, the pilin gene of only one representative isolate was included. The ClustalW [86] plugin in Geneious v5.4 [87] was used to align nucleotide sequences. Bayesian phylogenetic (BP) analyses were conducted using MrBayes v3.1.2 [88] with 2 chains of 2 million generations each using the GTR + I + Γ model. Trees were sampled every 200 generations, and the first 5,000 trees (10%) were discarded as burnin for each chain prior to generating the extended majority rule consensus tree. Maximum likelihood (ML) analyses were conducted using RAxML v7.0.3 [89] using the rapid BS algorithm [90] with 1,000 bootstraps and the GTR + I + Γ model.

**Figure 2:**
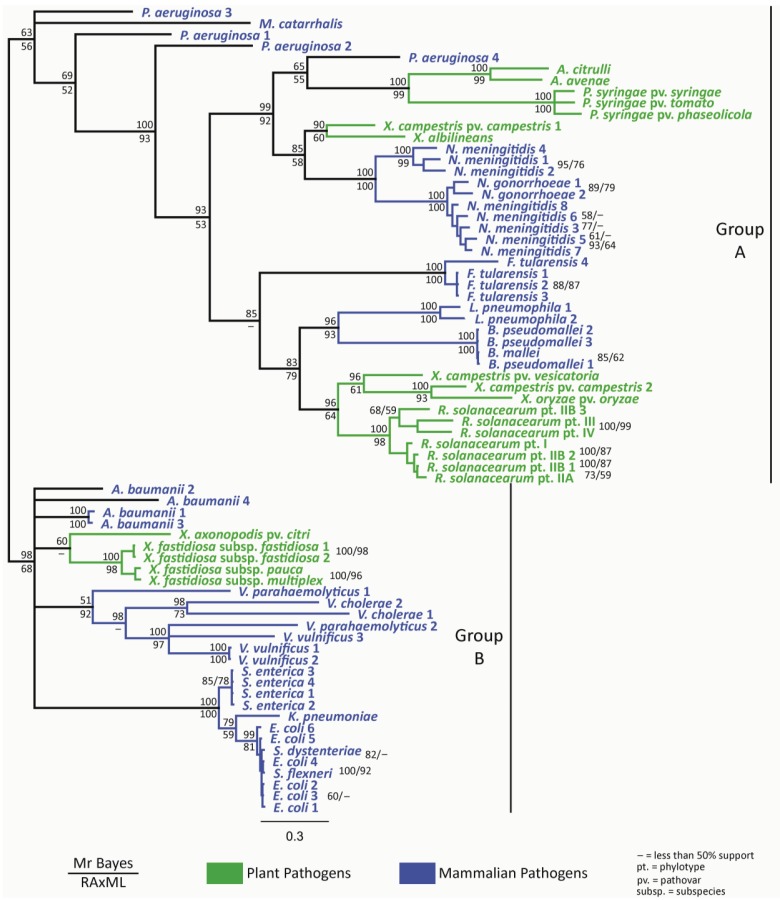
Phylogeny of the unique *pilA* (pilin) DNA sequences (n = 71) found among the complete genomes of plant and mammalian bacterial pathogens deposited in GenBank (Table S1, supplementary material) as inferred by Bayesian phylogenetic (BP) analyses. Nodes with <50% support were collapsed. BP posterior probabilities and maximum likelihood (ML) bootstrap values are presented above and below each node, respectively. Dashes indicate relationships not supported based on ML analyses. Group A contains bacteria with type IVa pili and Group B contains bacteria with type IVb pili. Plant pathogens are in green, and mammalian pathogens are in blue.

Our phylogenetic analyses distinguish between the two pilin groups previously defined for mammalian pathogens, namely group A and group B (type IVa and type IVb pilins; see above). Mammalian pathogens identified as belonging to group A (*P. aeruginosa, Neisseria* spp. and *Moraxella* spp.) and group B (*E. coli, S. enterica* and *V. cholerae*) [5,60] are separated into two major clades in our phylogeny (Figure 2). Pilin gene sequences from plant pathogenic bacteria were found to vary widely, but they do not form a group that is phylogenetically distinct from mammalian pathogens. Moreover, they can be clearly categorized as belonging to one of the two separate clades (Figure 2). In other words, the phylogenetic clades supported in our study can be used to infer the T4P group identity of the analyzed plant pathogens. The majority of plant pathogenic bacteria cluster with group A, which is the most diverse of the two pilin groups.

A trend was observed where plant pathogen isolates from the same genus or species belong to the same distinct clade, which is also true for mammalian pathogens. So, as might be expected, the pilin gene from one isolate is generally more similar to that of an isolate from the same genus or species than from another genus or species. There are two main exceptions to this trend. One exception is the *Xanthomonas* genus, where the four *Xanthomonas* species represented here do not group together in any clear way, showing that the *pilA* sequence does not clearly differentiate members of this genus. Specifically for *Xanthomonas campestris* pv. *campestris*, the two available *pilA* alleles from different isolates of the same pathovar belong to different clades. Xanthomonads are by far the most diverse group in terms of *pilA* sequence examined here, and this probably reflects the wide range of plant hosts infected by different *Xanthomonas* species and pathovars and the high genetic diversity that occurs among members of this genus, particularly within *X. campestris* pv. *campestris* [91]. However, most intriguing was the finding that the pilin gene sequence from the *Xanthomonas axonopodis* isolate used in this study clustered in pilin group B, in contrast to the other xanthomonad pilin genes that clustered within group A.

The other exception to this trend is the genus *Pseudomonas.* Pilin genes from the plant pathogen *Pseudomonas syringae* and the mammalian pathogen *Pseudomonas aeruginosa* do not cluster together by genus in phylogenetic analyses, but isolates within each species do cluster together. This indicates the role that host specificity can play in pilin sequence variability. Because T4P are highly involved in virulence, it may be that *pilA* is important for host adaptation, causing this gene to be fairly divergent in members of the same genus because of the diversity among host organisms.

Another interesting finding from these phylogenetic analyses was that the *X. axonopodis* isolate and *Xylella fastidiosa* isolates were the only plant pathogenic bacteria belonging to the group B pilin clade. *X. axonopodis* and *X. fastidiosa* are predominantly pathogens of woody plants, while the other plant pathogens assessed here (which all possess a group A pilin sequence) tend to infect herbaceous plants. Further experimentation is necessary to understand the biological function and significance of the different types of plant pathogenic bacterial pilin sequences delineated in these analyses and determine the effects of host specificity on T4P.

## 4. The Role of T4P in Pathogenicity of Plant Pathogenic Bacteria

In contrast to the well-established role played by T4P in pathogenicity of several animal pathogenic bacteria, the role of T4P in pathogenicity of plant pathogenic bacteria is poorly understood. In plant pathogens, the contributions of T4P to virulence have been investigated mainly in vascular pathogens, particularly in those possessing the ability to colonize and spread via the plant xylem vessels. It has been proposed that T4P may contribute to bacterial colonization and spread in the xylem through cell attachment, biofilm formation and twitching motility. Nevertheless, other reports have also demonstrated the involvement of T4P in epiphytic fitness and, recently, in virulence of non-vascular plant pathogenic bacteria. In this section we summarize the current knowledge of the role of T4P in the interactions between plant pathogenic bacteria and their host plants as well as in important fitness properties.

### 4.1. Ralstonia solanacearum

Although twitching motility was described in the mid-1970s [92], and despite a few reports on T4P of plant pathogenic bacteria during the 1980s and 1990s, the first study to demonstrate a substantial role of twitching motility in virulence of a vascular plant pathogenic bacterium was only published in 2001 [93]. This study was of the soil-borne bacterium *Ralstonia solanacearum*, which is widely distributed in tropical, subtropical and warm temperate regions. It causes bacterial wilt disease in a wide range of plants, infecting more than 200 plant species that belong to more than 50 botanical families [94]. *R. solanacearum* invades plant roots, colonizes the xylem vessels and spreads rapidly to the upper parts of the plant via the vascular system. Typical symptoms of the disease include browning of the xylem, foliar yellowing and lethal wilting. In the aforementioned pioneer study on plant pathogenic bacterium T4P, Liu and colleagues observed twitching motility in agar plates by microscopically viewing the fringe surrounding growing colonies. Twitching motility was abolished in *pilQ* and *pilT R. solanacearum* mutants, which also induced slower and less severe wilting symptoms in tomato relative to the wild type [93].

The same research group further studied the role of T4P by creating *pilA* mutants of *R. solanacearum* [95]. These mutants were affected in many pathogenicity-related phenotypes including reduced virulence in tomato plants, reduced autoaggregation and biofilm formation in culture, and lack of attachment ability to tobacco cells in culture suspensions and to tomato roots. The *pilA* mutants were also not competent for transformation, illustrating for the first time the multiple functions of T4P in a phytopathogen.

In their first report, Liu and colleagues noted that twitching motility in *R. solanacearum* was only observed during the first day of growth on agar plates and was no longer detected after two days [93]. Later, Kang and colleagues [95] showed that *pilA* gene expression was reduced at high cell densities in the wild-type strain, while mutants in the transcriptional regulator PhcA were able to keep high levels of *pilA* expression and twitching motility at high cell densities. The response regulator PehR, which is repressed by PchA, positively influences *pilA* expression. Cell density-dependent regulation of twitching motility may be important for the life cycle of *R. solanacearum*, which lives in the soil and inside the vascular system of the plant host, where it causes disease by forming biofilm and obstructing water flow inside the plant. While during the soil-inhabiting phase swimming motility by means of flagella may be crucial to respond to and move towards gradients of seed/root exudates and to start the infection process, once inside the confined environment of the xylem vessels, twitching ability may be important to allow the bacterium to spread and colonize other parts of the infected plant and overcome nutrient limitations [95].

### 4.2. Xylella fastidiosa

Among plant pathogenic bacteria, *Xylella fastidiosa* has been the most investigated in terms of T4P functions. *X. fastidiosa* is a xylem-limited, non-flagellated bacterium that causes diseases in many crops including grape, citrus, peach, plum, almond [96] and blueberry [97]. This bacterium, found mainly in the warmer regions of the Americas, causes important diseases such as Pierce's Disease (PD) of grapevines, which is prevalent in the US, and citrus variegated chlorosis (CVC) in citrus, occurring mainly in Brazil. *X. fastidiosa* forms biofilms inside plant xylem vessels, and this is believed to be responsible for obstruction of water and nutrient flow to the rest of the plant, causing disease symptoms such as leaf scorch and chlorosis, among others. The bacterium is transmitted among plants by sharpshooter insects that feed on the xylem fluid and carry the bacterium in their mouth parts. Once injected into the xylem, the bacterium moves acropetally with the xylem fluid flow, and basipetally against the flow via twitching motility [98].

That *X. fastidiosa* produces T4P was evidenced by *in silico* analysis of the full genome, where 25 putative *pil* genes were shown to be present [99], and proteomic analysis that detected PilY1, PilT, and other T4P components [100]. Confirmation that *X. fastidiosa* can move against the direction of fluid flow was elegantly proven by experiments using microfluidic flow chambers (MFCs) that mimic the plant xylem vessels [98]. Upstream twitching motility explained the development of symptoms basipetally from the point of inoculation. Using MFCs, it was shown that the substrate adhesion force exerted by the wild-type strain (∼147 pN) far exceed the shear force of media flow (∼80 fN) [98,101], assuring that cells are powerful enough to move against the xylem fluid flow.

**Figure 3:**
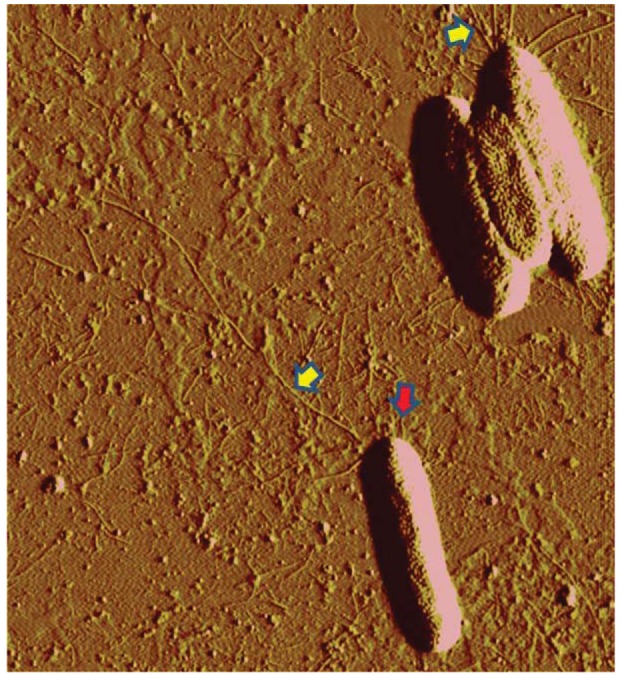
Atomic force micrograph of *Xylella fastidiosa* “Temecula” wild-type cells. Yellow arrows indicate T4P, while red arrows indicate shorter type I pili. Both types of pili are present at the same cell pole.

In *X. fastidiosa*, 1.0 to 5.8 μm long T4P are positioned at one of the cell's poles, while in the same pole, shorter (0.4 to 1.0 μm long) type I pili (T1P) are also present [98,102] (Figure 3). Mutational analysis of a T4P gene cluster showed that mutants in *pilB* and *pilT* [98], as well as in *fimT, pilX* and *pilO* [102], did not form T4P and were non-motile. Some of these mutants were not able to colonize upstream regions of grapevines [98]. Infection studies of grape plants in the greenhouse showed that T4P defective mutants were still infective, although symptoms were delayed and localized at the point of inoculation and did not spread along the vines [103]. Mutants in *pilY1*, believed to produce an adhesin located at the tip of T4P, showed reduced motility [102,104]. MFC studies showed that the speed of twitching motility against the flow of *pilY1* defective mutants was one third of that of the wild type [104]. These data suggest that lack of PilY1 may cause cells to “slip”, compromising efficient attachment to surfaces and slowing the rates of twitching motility. In the same study, a mutant in T1P moved seven times faster than the wild type. T1P is important for efficient attachment to surfaces, and seems to restrict the speed of T4P-mediated twitching motility in *X. fastidiosa* [104].

Several *X. fastidiosa* T4P mutants showed increased biofilm production [102], contrary to the concept that T4P contribute to the biofilm formation process. These results could be explained by a higher density of cells starting biofilms, achieved by a stronger attachment to surfaces by T1P in the absence of T4P. In other words, longer T4P likely disturb T1P contact with surfaces; therefore, in T4P deficient mutants, T1P can promote a stronger attachment to the surface and a consequent enhanced biofilm formation relative to the wild type [105].

Several proteins have been shown to have a regulatory role in function of T4P in *X. fastidiosa.* One example is TonB1, a protein located at the cytoplasmic membrane that is known to be involved in generating proton motive force for transport of iron-siderophore complexes and vitamin B12 across the outer membrane. A *tonB1* mutant lost twitching motility although T4P was still produced [106]. This mutant also showed reduced biofilm formation *in vitro* and caused reduced PD symptoms in grapevines [106]. It was hypothesized that TonB1 may be involved in providing energy for import of molecules into the cell that may be involved in twitching motility, other than the molecules involved in T4P assembly. PilS/PilR is a two-component regulatory system that senses environmental signals by the sensor protein PilS, which later phosphorylates PilR that in turn regulates several processes, including production of T4P. *pilR* mutants of *X. fastidiosa* are non-motile and do not form T4P [102]. *X. fastidiosa* was also shown to have a *pil-chp* operon homolog to chemosensory systems in other bacteria [107]. One of the genes in this operon is *pilL*, which is homologous to the transmembrane chemoreceptor *cheA* in *E. coli.* A *X. fastidiosa pilL* mutant was shown to lose twitching motility, though it still formed T4P. This mutant also possessed decreased biofilm formation ability and induced delayed and less severe symptoms in grapevines relative to the wild type. The authors conclude that the chemosensory system in *X. fastidiosa* contributes to virulence, probably by regulating T4P function [107].

Different chemical compounds have been shown to affect twitching motility in *X. fastidiosa*. Removal or reduction of BSA from solid media was shown to increase movement in more than a dozen *X. fastidiosa* strains isolated from grape in different geographic areas [108]. The cell-to-cell signaling system mediated by the diffusible signal factor (DSF) is known to regulate several virulence traits in *X. fastidiosa* [109], including biofilm formation and production of hydrolytic enzymes. A mutant defective in production of DSF showed faster twitching motility than the wild type, while addition of purified DSF to the media greatly reduced twitching motility in the wild type [110]. DSF was shown to positively regulate the T1P gene *fimA* [111]; therefore, the increase in surface attachment by T1P may explain the observed negative effect on twitching motility by DSF, since a connection between T1P and reduction of twitching motility was previously established [104]. These results support the hypothesis that DSF modulates twitching motility and are in agreement with the main function proposed for DSF, which is to impact the transition between plant and insect host lifestyles [112], when cells have to choose between exploring new environments or attaching strongly to surfaces. Regarding metallic ions, iron is an environmental signal for modulating the transcriptional control of T4P genes during *X. fastidiosa* colonization: growth under low iron conditions was shown to increase the expression of several *pil* genes [113]. Increases in calcium concentration were shown to enhance twitching motility in agar plates and movement speed inside MFCs [114].

Several studies used gene expression analysis to dissect factors involved in control of *pil* gene expression. Growth of *X. fastidiosa* in grape xylem fluid led to an upregulation of genes involved in twitching motility such as *fimT, pill, pilU* and *pilY1* [115]. Other studies showed that the PD1926 open reading frame, situated in the *pil* cluster and suggested to be involved in T4P formation, was under negative control by the alternate sigma factor AlgU [116], while it was positively controlled by GacA [117]. The role of PD1926 on twitching motility has not yet been proven. The lack of the global ^σ54^ regulator RpoN was shown to downregulate the expression of one out of the five *pilA* paralogues in a non-pathogenic strain of *X. fastidiosa* [118].

No role for T4P has been assigned in the interactions of *X. fastidiosa* with insect vectors. *pilB* and *fimA/pilO* mutants were not affected in attachment to polysaccharides present in the vector mouth nor insect foregut extracts [119]. Moreover, when cells were grown in a media that facilitates acquisition by insects, *pilY1* was downregulated, while genes involved in other attachment structures such as T1P were upregulated [120]. These observations suggest that T4P are not important for the interaction between *X. fastidiosa* and insect vectors. Accordingly, ongoing research is showing that T4P mutants are not impaired in bacterial transmission by insects to plant host [121].

### 4.3. Acidovorax citrulli

*Acidovorax citrulli* (formerly *Acidovorax avenae* subsp. *citrulli)* is a Gram-negative bacterium that infects different plants of the Cucurbitaceae family, mainly watermelon and melon [122]. Young seedlings and fruits are extremely susceptible to this pathogen, with fruit blotch and seedling blight, respectively, being the typical symptoms caused by this bacterium. *A. citrulli* is able to colonize and spread through vascular tissues of host plants [37,123], and it seems that this ability is important for induction of seedling blight and death, at least in melon plants [37].

**Figure 4:**
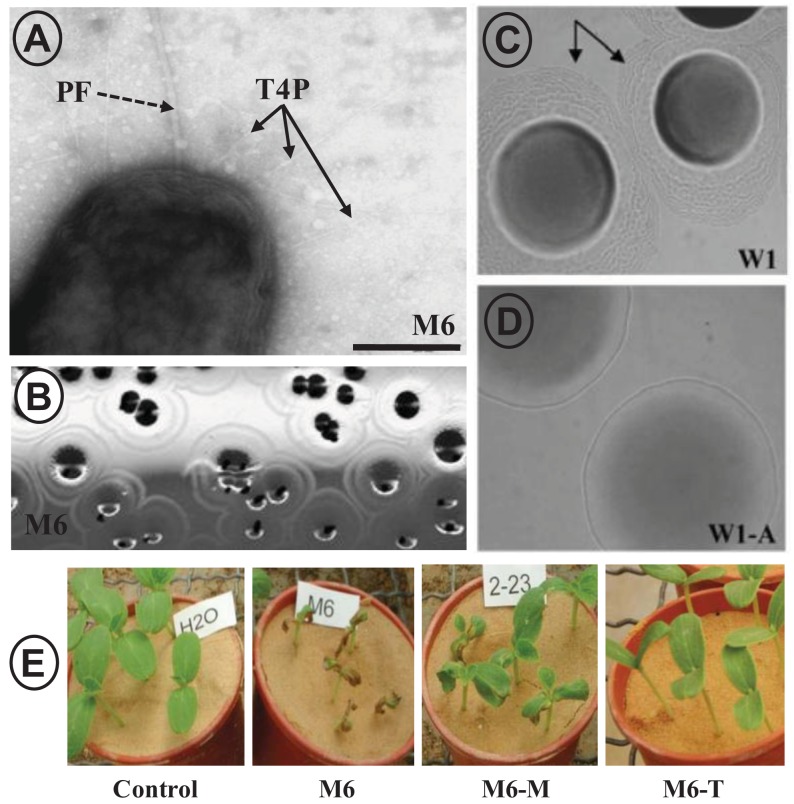
T4P in *Acidovorax citrulli*, causal agent of bacterial fruit blotch and seedling blight of cucurbit plants. (**A**) Transmission electron microscopy of wild-type strain M6 following growth for 48 h on nutrient agar (NA) plates. Solid and dashed arrows indicate T4P and polar flagellum (PF), respectively (bar = 0.5 μm); (**B**) Typical twitching halos surrounding bulk colonies of strain M6 after 96 h of growth on NA; seen by the naked eye; (**C**) Twitching halos seen around colonies of strain W1 by light microscopy after 96 h of growth on NA; (**D**) Halos are not detected around colonies of a W1 *pilA* mutant (strain W1-A; lacking T4P and twitching motility) grown under similar conditions as described for W1 in C; (**E**) Effects of T4P deficiency on virulence of *A. citrulli*, assessed by seed-transmission assays with melon cv. Ophir. Seeds were incubated with the different strains (at 10^6^ cfu/mL), and sowed in pots containing sand that were kept in a greenhouse at 25–28 °C. Pictures were taken 8 days after sowing. M6, wild type; M6-M, *pilM* mutant; M6-T, *pilT* mutant; control, seedlings from non-inoculated seeds. Figure composed from figures published by Bahar and colleagues [37].

Individual mutations in *pilM*, *pilA* and *pilT* genes revealed that *A. citrulli* depends on the T4P machinery for twitching motility on semisolid media (shown for *pilA* in Figure 4). T4P were also shown to be required for the formation of wild-type levels of biofilm on plastic and glass surfaces [37]. MFC studies with TFP deficient mutants of *A. citrulli* further emphasized the importance of T4P for xylem colonization. Under the pressure of shear force from medium flowing through the microscopic channels, T4P were shown to be crucial for surface attachment. T4P mutants were unable to form biofilm in this environment, while wild-type cells formed dense biofilms that covered the whole surface of the chambers within 48–96 h (supplementary movie 2) [124]. Similar to what was shown for *X. fastidiosa*, T4P also facilitate downstream migration of *A. citrulli* via twitching motility [37,124]. Consistent with these *in vitro* assays showing the importance of T4P for various abilities of *A. citrulli*, T4P mutants were severely impaired in virulence on melon seedlings (Figure 4) following seed or stem inoculation. In both assays, significant differences in the percentage of dead seedling were observed between T4P mutants and the wild type. Moreover, *in planta* downward migration assays also supported the results from MFC experiments showing that T4P are important for efficient migration in the plant vascular system [37]. In contrast, no significant differences in symptom induction ability and growth *in planta* have been observed between a *pilM* mutant that does not produce T4P and the wild-type strain, following foliage inoculation of melon plants by both dip and spray methods [125]. These findings suggest that, in contrast to their importance of T4P for vascular infection, T4P and twitching motility may not have a crucial role in local, foliar infection by *A. citrulli*. Whether T4P significantly contribute to fruit infection by this pathogen is a subject that has not yet been investigated.

Recent characterization of polar flagellum mutants of *A. citrulli* revealed that polar flagella contribute to the virulence of this bacterium following seed, stem and foliage inoculation [126]. Interestingly, polar flagellum mutants were not affected in biofilm formation ability, but they did show significantly reduced twitching motility in agar plates relative to the wild type. Transmission electron microscopy revealed that the polar flagellum mutants were not affected in their ability to produce T4P [126]. On the other hand, T4P mutants were previously shown to possess significantly altered swimming ability in comparison with the wild type: while the *pilM* mutant, lacking T4P, swims faster than the wild type, the hyperpiliated *pilT* mutant is significantly impaired in swimming motility [37]. These findings and the fact that T4P and polar flagella co-locate at one of the cell poles of the bacterium suggest that T4P might mechanically disturb swimming via the polar flagellum. Nevertheless, other types of interactions between these two organelles, including at regulatory levels, cannot be discarded.

### 4.4. Xanthomonas Species

The *Xanthomonas* genus contains more than 100 different plant pathogens that cause disease in over 300 different plant species. Surprisingly, the contribution of T4P to virulence and fitness has been studied in few xanthomonads. Some xanthomonads are vascular pathogens, while others infect the plant tissue locally. T4P mutants of the vascular pathogens *X. oryzae* pv. *oryzae* (Xoo; bacterial leaf blight of rice) and *X. campestris* pv. *campestris (Xcc;* black rot disease of cruciferous plants) were shown to possess reduced virulence relative to their parental strains. *pilQ* mutants of *Xoo* lack twitching motility, are deficient in biofilm formation and possess reduced virulence [127-129], while a *pilA* mutant of *Xcc* was shown to be negatively affected in surface motility and, to some extent, in virulence [130].

In contrast to *Xoo* and Xcc, *X. campestris* pv. *vesicatoria* (*Xcv*; bacterial spot disease of tomato and pepper) does not possess vascular colonization ability. Ojanen-Reuhs and colleagues [131] characterized a *fimA* (pilin) mutant of *Xcv* and showed that, despite clear impairments of this mutant in aggregate formation, adherence to leaf trichomes and resistance to UV radiation, no differences were observed between this mutant and the wild-type strain in symptom induction and growth in host plants following both infiltration and foliage spray inoculations. Van Doorn and colleagues [132] showed that *X. hyacinthi* cells (yellow disease of hyacinth) and purified type T4P attach to stomata of hyacinth leaves and suggested a role for T4P in the initial stages of infection of hyacinth leaves by this pathogen; however, this hypothesis was not further examined.

Significant contribution of T4P to virulence was recently demonstrated for the non-vascular pathogen *X. oryzae* pv. *oryzicola* (*Xoc*; bacterial leaf streak of rice) following a virulence screen of a collection of transposon mutants. Transposon insertions that yielded *Xoc* mutants with reduced virulence were identified in *pilY, pilQ, pilM, pilZ* and *pilT* genes. While for most of these mutants the reduction in virulence was slight to moderate, the *pilT* mutant was severely affected in this feature. [133]. To the best of our knowledge, *Xoc* is the only nonvascular xanthomonad for which a significant contribution of T4P to virulence has been demonstrated to date.

### 4.5. Pseudomonas Species

The *Pseudomonas* genus contains several plant pathogenic species of which *P. syringae*, comprising more than 50 different pathovars, is the most economically important [134]. As with xanthomonads, the involvement of *P. syringae* T4P in plant-pathogen interactions has been investigated in very few pathovars, with most of the reports being published over a decade ago.

*P. syringae* pv. *phaseolicola (Psp;* halo blight of common bean) was the first *P. syringae* pathovar to be investigated in terms of the role of T4P in plant-pathogen interactions. Romantschuk and colleagues [135] compared the leaf attachment ability of different *Psp* strains with natural variation in piliation. Leaf attachment assays showed a positive correlation between the degree of piliation and leaf adherence when bacteria were spray-inoculated onto the leaf surface. In addition, piliated bacteria appeared to attach more specifically to leaf stomata, while non-piliated bacteria attached more uniformly to the leaf surface, suggesting a role of T4P in attachment to stomata, as also inferred by the aforementioned study of Van Doorn and colleagues [132] with *X. hyacinthi.* When the bacterial inoculum was introduced by either stabbing or infiltration into bean leaves, no differences in symptom severity were observed among the different strains, suggesting a negligible contribution of T4P to virulence of this pathogen at post-penetration stages. However, clear differences among strains with different levels of piliation were observed when bacteria were spray-inoculated onto the leaf surface. In these assays, piliation and symptom induction ability correlated positively, while non-piliated strains were unable to induce visible symptoms, suggesting an important role of T4P for invasion into the leaf tissue [135].

Piliation was also shown to be important for initial adhesion and colonization of leaves by another common bean pathogen, *Pseudomonas syringae* pv. *syringae* (*Psy;* brown spot disease) [136]. However, no pathogenicity assays to assess the contribution of T4P to virulence of *Psy* were reported. Characterization of a *pilA* non-piliated mutant of *P. syringae* pv. *tomato* (*Pto;* bacterial speck disease of tomato) revealed that T4P contribute to UV tolerance in laboratory conditions and epiphytic fitness *in planta.* However, no differences were observed between the *pilA* mutant and the wild-type strain in their ability to induce disease symptoms on susceptible tomatoes following direct infiltration or foliage spray inoculation. In contrast to the aforementioned common bean pathovars, only small and apparently non-consistent differences were found between the *pilA* mutant and the *Pto* wild type in leaf attachment assays [137].

Recently, the role of T4P in plant-pathogen interactions was also examined in *Pseudomonas syringae* pv. *tabaci* (*Pta;* wildfire disease of tobacco) by Taguchi and Ichinose [138]. Surprisingly, the authors reported that *Pta* mutants impaired in *pilA* and *pilO* retained T4P-mediated twitching motility, although swimming and swarming motilities were attenuated in both mutants. The mutants were also shown to possess reduced biofilm formation ability *in vitro* relative to the wild type. In pathogenicity assays, no differences between the T4P mutants and the wild type were observed when inocula were directly infiltrated into leaves. However, the T4P mutants were shown to be significantly injured in terms of symptom induction and bacterial multiplication following foliage dip inoculation. Interestingly, the T4P mutants also had reduced expression of *hrp* genes and were slower than the wild type in hypersensitive response (HR) induction in non-host plants, suggesting co-regulation of T4P and the type III secretion machinery in this pathogen [138].

## 5. Conclusions

Investigations into the involvement of T4P in the interactions of plant pathogenic bacteria with their plant hosts are in the early stages, with most knowledge on this subject being accumulated only during the last decade. For some plant pathogenic bacterial species, it has been demonstrated that T4P play an important role in pathogenicity. T4P are important virulence factors in most evaluated vascular plant pathogenic bacteria. Findings from different vascular pathogen-host systems, summarized here, indicate that twitching motility and biofilm formation promoted by T4P are crucial for spread and colonization inside host xylem vessels. Recently, a significant contribution of T4P to virulence has been reported for a few non-vascular bacteria (like *X. oryzae* pv. *oryzicola* and *P. syringae* pv. *tabaci*). In some other non-vascular plant pathogenic bacteria, although T4P were not found to contribute to virulence, they were shown to promote epiphytic ability and survival under different stresses. Though not directly related to virulence, it is logical to assume that these features are important for bacterial infection under field conditions. Moreover, the possibility that T4P directly contribute to virulence in these pathogens cannot be completely discarded, as this issue has not been thoroughly investigated, and it is known that many virulence factors in plant pathogenic bacteria possess a subtle phenotype that may not be detected in artificial inoculation conditions [139].

Genetic and genomic studies of plant pathogenic bacteria have revealed that T4P genes are conserved and homologous with those of animal pathogenic bacteria. As shown in this study, phylogenetic analyses of pilin sequences do not discriminate between plant and animal pathogenic bacteria. T4P of plant- and animal-associated bacteria are also functionally conserved. For most plant pathogenic bacteria, however, there are many open questions regarding the importance of the functions and processes conferred by T4P to virulence and fitness. Other relevant questions deserving further attention include: Under which conditions and at which stages of infection are T4P genes expressed? Which genetic and environmental factors regulate the expression of T4P genes and T4P function? How does T4P expression and function interact with other virulence determinants like polar flagella, extracellular polysaccharides, type III secretion, multidrug resistance and others to promote plant infection and disease? Elucidation of these questions will contribute to a better understanding of the role played by T4P in the interactions between plant pathogenic bacteria and plants. Due to the importance of these organelles for colonization, survival and virulence (depending on the study organism), advances in this area may lead to the development of new strategies to combat these pathogens for the benefit of agriculture.

## Supplementary Material

**Table S1:** Bacterial pathogen complete genomes from GenBank used to obtain pilA DNA sequences used in pilA phylogeny (Figure 2).

**Phylogeny Name a**	**Genus**	**Species**	**Rank under species b**	**Isolate**	**Accession Number**
***A. avenae***	*Acidovorax*	*avenae*	-	ATCC 19860	CP002521
***A. citrulli***	*Acidovorax*	*citrulli*	-	AAC00-1	CP000512
***A. baumanii* 1**	*Acinetobacter*	*baumanii*	-	ACICU	CP000863
***A. baumanii* 2**	*Acinetobacter*	*baumanii*	-	ATCC 17978	CP000521
***A. baumanii* 3**	*Acinetobacter*	*baumanii*	-	TCDC-AB0715	CP002522
***A. baumanii* 4**	*Acinetobacter*	*baumanii*	-	AB0057	CP001182
***B. mallei***	*Burkholderia*	*mallei*	-	NCTC 10247	CP000548
***B. pseudomallei* 1**	*Burkholderia*	*pseudomallei*	-	1106a	CP000572
***B. pseudomallei* 2**	*Burkholderia*	*pseudomallei*	-	MSHR346	CP001408
***B. pseudomallei* 3**	*Burkholderia*	*pseudomallei*	-	668	CP000570
***E. coli* 1**	*Escherichia*	*coli*	-	55989	CU928145
***E. coli* 2**	*Escherichia*	*coli*	sv. O111:H	11128	AP010960
***E. coli* 3**	*Escherichia*	*coli*	-	UMNK88	CP002729
***E. coli* 4**	*Escherichia*	*coli*	sv. O157:H7	TW14359	CP001368
***E. coli* 5**	*Escherichia*	*coli*	-	UM146	CP002167
***E. coli* 6**	*Escherichia*	*coli*	sv. O83:H1	NRG 857C	CP001855
***F. tularensis* 1**	*Francisella*	*tularensis*	*holarctica*	FTNF002-00	CP000803
***F. tularensis* 2**	*Francisella*	*tularensis*	*holarctica*	OSU18	CP000437
***F. tularensis* 3**	*Francisella*	*tularensis*	*mediasiatica*	FSC147	CP000915
***F. tularensis* 4**	*Francisella*	*tularensis*	*novicida*	U112	CP000439
***K. pneumoniae***	*Klebsiella*	*pneumoniae*	-	342	CP000964
***L. pneumophila* 1**	*Legionella*	*pneumophila*	-	2300/99 Alcoy	CP001828
***L. pneumophila* 2**	*Legionella*	*pneumophila*	-	Corby	CP000675
***M. catarrhalis***	*Moraxella*	*catarrhalis*	-	RH4	CP002005
***N. gonorrhoeae* 1**	*Neisseria*	*gonorrhoeae*	-	NCCP11945	CP001050
***N. gonorrhoeae* 2**	*Neisseria*	*gonorrhoeae*	-	TCDC-NG08107	CP002440
***N. meningitidis* 1**	*Neisseria*	*meningitidis*	sg. C	FAM18	AM421808
***N. meningitidis* 2**	*Neisseria*	*meningitidis*	-	G2136	CP002419
***N. meningitidis* 3**	*Neisseria*	*meningitidis*	-	M01-240355	CP002422
***N. meningitidis* 4**	*Neisseria*	*meningitidis*	-	WUE2594	FR774048
***N. meningitidis* 5**	*Neisseria*	*meningitidis*	-	MC58	AE002098
***N. meningitidis* 6**	*Neisseria*	*meningitidis*	-	8013	FM999788
***N. meningitidis* 7**	*Neisseria*	*meningitidis*	sg. A	Z2491	AL157959
***N. meningitidis* 8**	*Neisseria*	*meningitidis*	-	M01-240149	CP002421
***P. aeruginosa* 1**	*Pseudomonas*	*aeruginosa*	-	LESB58	FM209186
***P. aeruginosa* 2**	*Pseudomonas*	*aeruginosa*	-	PA7	CP000744
***P. aeruginosa* 3**	*Pseudomonas*	*aeruginosa*	-	PAO1	AE004091
***P. aeruginosa* 4**	*Pseudomonas*	*aeruginosa*	-	UCBPPPA1 4	AY049071
***P. syringae* pv. *syringae***	*Pseudomonas*	*syringae*	pv. *syringae*	B728a	CP000075
***P. syringae* pv. *tomato***	*Pseudomonas*	*syringae*	pv. *tomato*	DC3000	AE016853
***P. syringae* pv. *phaseolicola***	*Pseudomonas*	*syringae*	pv. *phaseolicola*	1448A	CP000058
***R. solanacearum* pt. IIB1**	*Ralstonia*	*solanacearum*	pt. IIB	IPO1609	CU914168
***R. solanacearum* pt. IIB2**	*Ralstonia*	*solanacearum*	pt. IIB	MolK2	CU861906
***R. solanacearum* pt. IIA**	*Ralstonia*	*solanacearum*	pt. IIA	CFBP2957	FP885897
***R. solanacearum* pt. I**	*Ralstonia*	*solanacearum*	pt. I	GMI1000	AL646052
***R. solanacearum* pt. IIB3**	*Ralstonia*	*solanacearum*	pt. IIB	Po82	CP002819
***R. solanacearum* pt. III**	*Ralstonia*	*solanacearum*	pt. III	CMR15	FP885895
***R. solanacearum* pt. IV**	*Ralstonia*	*solanacearum*	pt. IV	PSI07	FP885906
***S. enterica* 1**	*Salmonella*	*enterica*	*enterica* sv. Choleraesuis	SC-B67	AE017220
***S. enterica* 2**	*Salmonella*	*enterica*	*enterica* sv. Heidelberg	SL476	CP001120
***S. enterica* 3**	*Salmonella*	*enterica*	*enterica* sv. Newport	SL254	CP001113
***S. enterica* 4**	*Salmonella*	*enterica*	*enterica* sv. Typhimurium	UK-1	CP002614
***S. dysenteriae***	*Shigella*	*dysenteriae*	-	Sd197	CP000034
***S. flexneri***	*Shigella*	*flexneri*	-	2002017	CP001383
***V. cholerae* 1**	*Vibrio*	*cholerae*	-	HE39	AFOQ01000009
***V. cholerae* 2**	*Vibrio*	*cholerae*	-	MJ-1236	CO001485
***V. parahaemolyticus* 1**	*Vibrio*	*parahaemolyticus*	-	RIMD 2210633	BA000031
***V. parahaemolyticus* 2**	*Vibrio*	*parahaemolyticus*	-	10329	AFBW01000001
***V. vulnificus* 1**	*Vibrio*	*vulnificus*	-	CMCP6	AE016795
***V. vulnificus* 2**	*Vibrio*	*vulnificus*	-	MO6-24/O	CP002469
***V. vulnificus* 3**	*Vibrio*	*vulnificus*	-	YJ016	BA000037
***X. oryzae.* pv. *oryzae***	*Xanthomonas*	*oryzae*	pv. *oryzae*	KACC10331	AE013598
***X. albilineans***	*Xanthomonas*	*albilineans*	-	GPE PC73	FP565176
***X. axonopodis.* pv. *citri***	*Xanthomonas*	*axonopodis*	pv. *citri*	306	AE008923
***X. campestris* pv. *campestris* 1**	*Xanthomonas*	*campestris*	pv. *campestris*	8004	CP000050
***X. campestris* pv. *campestris* 2**	*Xanthomonas*	*campestris*	pv. *campestris*	B100	AM920689
***X. campestris* pv. *vesicatoria***	*Xanthomonas*	*campestris*	pv. *vesicatoria*	85-10	AM039952
***X. fastidiosa* subsp. *fastidiosa* 1**	*Xylella*	*fastidiosa*	*fastidiosa*	Temecula	AE009442
***X. fastidiosa* subsp. *fastidiosa* 2**	*Xylella*	*fastidiosa*	*fastidiosa*	M23	CP001011
***X. fastidiosa* subsp. *multiplex***	*Xylella*	*fastidiosa*	*multiplex*	M12	CP000941
***X. fastidiosa* subsp. *pauca***	*Xylella*	*fastidiosa*	*pauca*	9a5c	AE003849

a Name in phylogenetic tree (Figure 2);

b Ranks under species: subspecies, phylotype (pt.), pathovar (pv.), serovar (sv.) and/or serogroup (sg.).

Supplementary File 1 ZIP-Document (ZIP, 15163 KB)
